# Structure of the Scientific Community Modelling the Evolution of Resistance

**DOI:** 10.1371/journal.pone.0001275

**Published:** 2007-12-05

**Authors:** REX Consortium

**Affiliations:** 1 INRA, France; Northeastern University, United States of America

## Abstract

Faced with the recurrent evolution of resistance to pesticides and drugs, the scientific community has developed theoretical models aimed at identifying the main factors of this evolution and predicting the efficiency of resistance management strategies. The evolutionary forces considered by these models are generally similar for viruses, bacteria, fungi, plants or arthropods facing drugs or pesticides, so interaction between scientists working on different biological organisms would be expected. We tested this by analysing co-authorship and co-citation networks using a database of 187 articles published from 1977 to 2006 concerning models of resistance evolution to all major classes of pesticides and drugs. These analyses identified two main groups. One group, led by ecologists or agronomists, is interested in agricultural crop or stock pests and diseases. It mainly uses a population genetics approach to model the evolution of resistance to insecticidal proteins, insecticides, herbicides, antihelminthic drugs and miticides. By contrast, the other group, led by medical scientists, is interested in human parasites and mostly uses epidemiological models to study the evolution of resistance to antibiotic and antiviral drugs. Our analyses suggested that there is also a small scientific group focusing on resistance to antimalaria drugs, and which is only poorly connected with the two larger groups. The analysis of cited references indicates that each of the two large communities publishes its research in a different set of literature and has its own keystone references: citations with a large impact in one group are almost never cited by the other. We fear the lack of exchange between the two communities might slow progress concerning resistance evolution which is currently a major issue for society.

## Introduction

During the last century, the generalised and intensive use of human-made chemical pesticides and drugs (including antimicrobial and antimalarial drugs, insecticides, herbicides, fungicides, nematicides, and miticides) has allowed significant progress in controlling major threats to human health and agriculture [1]–[3]. However, the resistance to drugs and pesticides in pathogenic organisms, disease vectors and agricultural pests has generally developed shortly after the introduction of new molecules often resulting in significant control failures [1], [4]. Antimicrobial drug resistance is an ever-increasing threat for public health. Since antibiotics came into general use in the 1950s, medical research has had to confront the recurrent evolution of resistance to most antibiotics used in hospitals against major microbial pathogens. For instance, a few years after the introduction of penicillin in 1943, strains of *Staphylococcus aureus* resistant to this antibiotic were detected in civilian hospitals [5]. Twenty years later, 80% of hospital *S. aureus* isolates were declared penicillin resistant [6]. More generally, the emergence of multidrug resistant bacterial strains has contributed to the continuous increase of hospital-acquired infections [7]. During the 20^th^ century, insecticide resistance in disease vectors and agricultural pests has also emerged as a problem. In 1986, Georghiou [1] reported the existence of about 500 insect species resistant to at least one insecticide, 100 resistant plant-pathogens and more than 45 herbicide-resistant weed species. The evolution of insecticide resistance in mosquitoes is a remarkable instance of rapid human-induced changes in pest populations. Dichloro-diphenyl-trichloroethane (DDT) was first introduced to control mosquitoes in 1946, and one year later the first resistant mosquito species, *Aedes tritaeniorhynchus* and *A. solicitans*, were detected. Currently, more than 100 mosquito species are known to be DDT resistant (cited by Hemingway et al. [8]).

This situation would not be necessarily problematic if pharmaceutical industries and agribusiness companies were able to stay one step ahead of pathogenic organisms and agricultural pests, i.e. to develop and market new products before resistance causes significant control failures. However, the rate at which resistance evolves in target organisms makes the development of new pesticides and drugs increasingly costly and difficult [9]–[11]. In addition, cross resistance between chemicals belonging to the same family often results in molecules becoming ineffective before they are used. So, not only must the chemical product be novel but its target must also be novel. Because the number of molecules that can be developed is necessarily limited, the efficacy of existing products should be protected for the long-term. In view of these considerations, modelling the evolution of resistance became a keystone approach in agricultural and medical research with the aim of identifying the best strategies to avoid or at least delay the development of resistance [3].

Models of resistance evolution (either mathematical models or computer simulations) consider the evolutionary forces governing the temporal dynamics of adaptive genes in populations subjected to strong directional selection. These forces (selection, drift, mutation and migration, to point out the majors) have been clearly identified [12] and are logically identical for virus, bacteria, fungi, plants and arthropods facing drugs or pesticides. Therefore, scientists modelling the evolution of resistance in different biological organisms would be expected to work together and interact, publish in the same scientific journals, quote the same scientific references, use similar modelling approaches and, ultimately, share the same basic management strategies to avoid the development of resistance.

**Figure 1:**
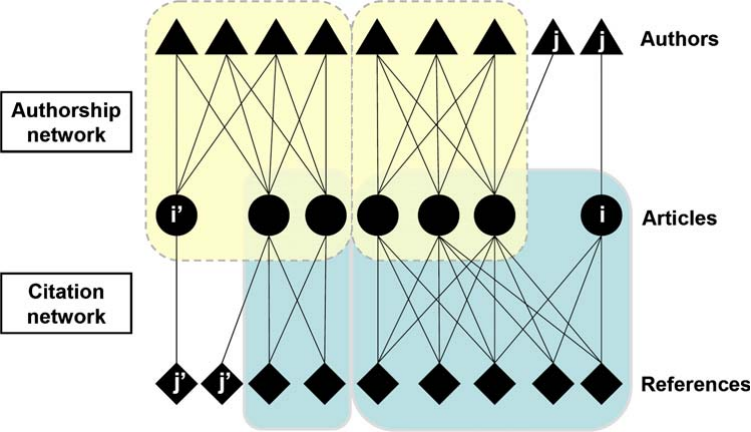
A schematic representation of the network analysis described in this study. The middle layer represents the research articles (circles) selected for the study. Upper and lower layers represent authors of the articles (triangles) and bibliographic references cited in the articles (diamonds), respectively. Linking the three layers together gives rise to two bipartite networks. The architecture of the authorship network (upper network) was analysed to assess the extent to which the scientists collaborated. The architecture of the citation network (lower network) was analysed to quantify to which extent the knowledge circulates among them. In this example, two distinct collaborative groups (yellow, dotted lines) establish their research from different sets of cited literature (blue, continuous lines). Authors having published once (j) and their corresponding articles (i) were removed from the authorship network. Likewise references that were cited only once (j') and the corresponding citing articles (i') were removed from the citation network. Articles i and i' were considered to be articles “isolated” from the authorship and citation networks, respectively.

Our aim was to test whether this is the case. We analysed co-authorship and co-citation networks using a database of 187 models of resistance evolution published from 1977 to 2006 (Table S1). These network analyses describe the extent to which scientists modelling resistance evolution collaborate and share their knowledge (Figure 1). We used historical, methodological and geographical criteria to interpret the structure of the scientific community.

## Results

### Database content

The bibliographic search in the CABs 1973–2006, Current Contents 1998–2006 and Medline 1950–2006 provided a dataset of 1,894 non redundant articles published in peer-reviewed scientific journals, dealing with resistance to pesticides or drugs. We removed all articles that did not deal with mathematical description or computer simulation of the temporal evolution of resistance. This resulted in a database containing 187 articles written by 321 different authors and citing a total of 4,154 bibliographic references. This database covered the range of all major drugs and pesticides: insecticidal proteins (39 articles), chemical insecticides (30), antibiotic drugs (29), herbicides (18), fungicides (15), antiviral drugs (14), antimalarial drugs (12), antihelminth drugs (10) and miticides (3); in addition there were 17 articles (hereafter referred to as ‘unspecific articles’) describing the evolution of resistance without reference to any specific class of pesticides or drugs.

The 187 articles were published between 1977 and 2006. More articles were published after than before 1995, mainly because of a larger number dealing with the evolution of drug resistance in bacteria and viruses. After 1995, publications concerning models of the evolution of resistance to chemical insecticides and to fungicides tended to be replaced by articles focusing on insecticidal proteins.

### Analysis of the authorship network

**Figure 2:**
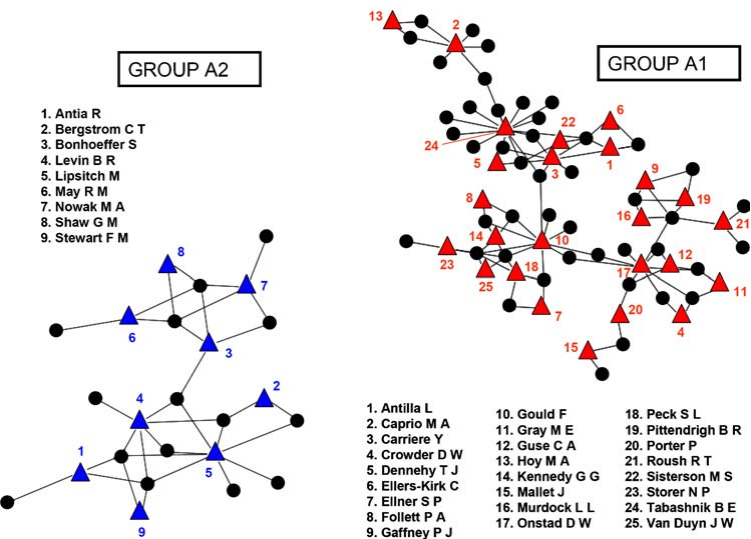
Largest components of the authorship network. Scientists (coloured triangles) are linked together through the articles (black circles) they have co-authored. The figure was obtained using the Tulip software [41]

Forty-nine of the 187 articles were written by scientists who were not authors of any other article included in the database. These 49 articles were classified as ‘isolated’ articles and were not included in the authorship network analysis. This analysis was therefore based on 138 articles with 87 authors (necessarily authors of at least two articles). The authorship network was fragmented into 28 components of various sizes: a large component including 45 articles and 25 authors (named group A1; Figure 2), a medium component including 15 articles and 9 authors (group A2; Figure 2) and 26 small components each including fewer than 6 articles and 5 authors (‘small groups’).

We investigated possible causes of the fragmentation of the authorship network into collaborative groups by characterizing each article of the database according to: the type of drug or pesticide considered; the type of target organism considered; the modelling approach used; the first author's geographical location; and the first author's academic discipline (Table 1). We then tested for statistical associations between these descriptive categories and the observed collaborative grouping. The distributions of descriptors in each category were differently associated within the groups A1 and A2 (Fisher's exact tests, *p*<10^−5^ for each category). Articles belonging to group A1 were predominantly written by North-American biologists who only used a population genetics approach to model resistance evolution to insecticides or insecticidal proteins in farm pests or farm diseases. One third of articles belonging to group A2 were written by European scientists and two thirds by North-American scientists; two thirds of these articles used epidemiological approaches to study the evolution of resistance in human parasites to antibiotic or antiviral drugs. Not surprisingly, more than two thirds of the authors were biologists, and a substantial proportion of the authors worked in medical institutes (Table 1). Both groups (A1 and A2) were too small for a clustering algorithm to be used.

**Table 1:** Within-group distribution of articles for the different descriptive categories.

Category	Descriptor	Percentage of Articles			
		A Groups		C Groups	
		A1	A2	C1	C2
Type of Drug or Pesticide	Antibiotic Drug	0.0	66.7	0.0	61.4
	Antihelminthic Drug	0.0	0.0	7.2	0.0
	Antimalarial Drug	0.0	6.7	8.7	0.0
	Antiviral Drug	0.0	20.0	0.0	31.8
	Fungicide	0.0	0.0	10.1	2.3
	Herbicide	0.0	0.0	13.0	0.0
	Insecticidal Protein	62.2	0.0	27.5	0.0
	Insecticide	26.7	0.0	21.0	0.0
	Miticide	2.2	0.0	1.4	0.0
	Unspecific	8.9	6.7	10.9	4.5
Type of Target Organism	Farm Pest or Disease	100.0	6.7	83.3	2.3
	Human Parasite	0.0	93.3	11.6	93.2
	Unspecific	0.0	0.0	5.1	4.5
Modelling Approach	Epidemiology	0.0	66.7	6.5	72.7
	Population Genetics	100.0	13.3	76.1	4.5
	Other	0.0	20.0	17.4	22.7
First Author's Location	Asia	0.0	0.0	8.7	2.3
	Europe	2.2	33.3	28.3	38.6
	North America	95.6	66.7	52.2	56.8
	Oceania	2.2	0.0	9.4	0.0
	South America	0.0	0.0	1.4	2.3
First Author's Discipline	Biology	95.6	73.3	86.2	36.4
	Economy	0.0	0.0	0.0	2.3
	Mathematics	4.4	6.7	7.2	13.6
	Medicine	0.0	20.0	6.5	47.7

For all categories, the distributions are significantly heterogeneous between groups A1 and A2, and between groups C1 and C2 (Fisher exact test, *p*<10^−5^ in both cases).

‘Small groups’ included a large range of articles. They addressed all types of drugs and pesticides, and used both modelling approaches: population genetics (49%) and epidemiology (24%). More than 80% of the articles describing resistance to herbicides, fungicides, and antihelminthic drugs belonged to these small authorship networks (Table S2). The characteristics of isolated articles were diverse (Table S2), and they were mostly studies on insect farm pests and insect disease vectors; they used both epidemiological and population genetics models.

### Analysis of the citation network

**Figure 3:**
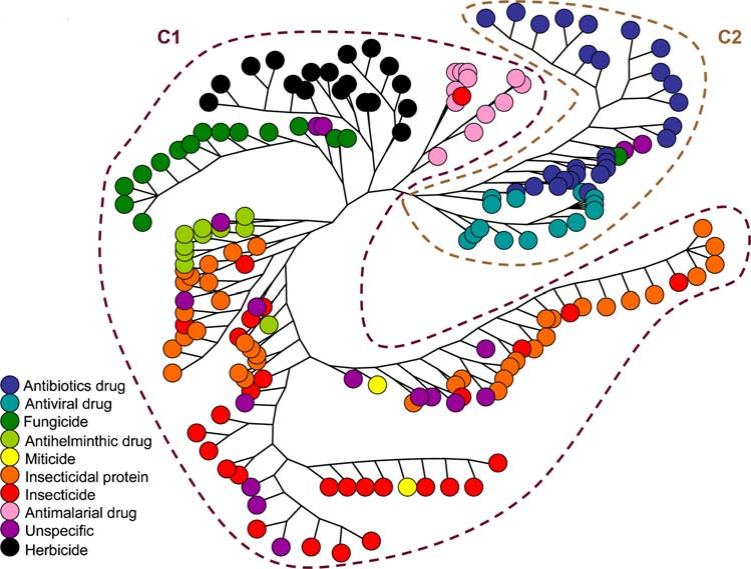
Hierarchical tree showing the structure of the citation network calculated using the ‘edge betweenness’ algorithm [13]. Due to space constraints, only the tree leaves corresponding to articles are depicted. Tree branches correspond to the splits of the network. The very first split produced two clusters called C1 and C2. Subsequent splits revealed divisions between seven subgroups within the C1 group. Tree leaf colours indicate the type of pesticide or drug considered by the articles.

A total of 4,154 references were cited by the 187 articles of the database; 3,297 were cited only once and were not included in the analysis. Five of the 187 articles of the database (further classified as ‘isolated articles’; see Figure 1) did not contain any list of references or did not share any reference with other articles of the database. These five articles were removed from the analysis. The citation network was hence composed of 182 articles and 857 citations. Unlike the authorship network, the citation network was fully connected. The clustering algorithm developed by Girvan and Newman [13] was used to investigate its structure: it organises the network in such a way that groups of densely connected nodes (here, articles and cited references) are separated from each other. The first split formed a group of 138 articles and 631 cited references (called C1; Figure 3) and a group of 44 articles and 226 cited references (called C2; Figure 3). The clustering into the groups C1 and C2 was statistically validated by the multiresponse permutation procedure: the citation dissimilarity between articles of the database was lower within than between groups C1 and C2 (A = 0.010, *p*<0.001, Table S3). Conversely, the dissimilarity of source articles among citations was statistically lower within than between groups C1 and C2 (A = 0.013, *p*<0.001, Table S3).

When applied to the unipartite projection of the citation network on articles (see material and methods for details on this projection), the clustering algorithm produced two major groups named U1 and U2; Table S4) which were strongly correlated with the C1 and C2 groups, respectively. About 97% of the C1 articles belonged to the U1 group, and about 93% of the articles in group C2 belonged to the U2 group. Five articles of the C1 group were classified in the U2 group, and all but one of them addressed resistance to antimalarial drugs. This suggests that the antimalaria articles were loosely associated with group C1.

We had included all types of references in the bipartite citation network, so references shared by articles belonging to the same citation group, whether C1 or C2, could be either theoretical studies on the evolution of resistance or articles describing the biology of target organisms. To circumvent the bias of common grouping resulting from shared biological references, we applied the clustering algorithm to the unipartite network: this network links two articles of the database if one of them cited the other. Since our database included only theoretical models, common grouping could then only be due to shared theoretical references. We found that this unipartite co-citation network also displayed two major groups, named M1 and M2 (Table S5), which were, like U1 and U2, strongly correlated with the C1 and C2 groups, respectively. About 91% of the C1 articles belonged to the M1 group, and about 86% of the articles of the C2 group belonged to the M2 group. Eleven articles of the C1 group were classified in the M2 group; they included nine focusing on resistance to antimalarial drugs. This confirmed that the antimalaria articles were loosely associated with the C1 group.

The distributions of four of the five categories of descriptors were different within the groups C1 and C2 (Fisher's exact tests, *p*<10^−5^). The location of the first author was the only category for which the distributions of the descriptors did not differ (at the 5% risk level) between the groups C1 and C2 (Fisher's exact test, *p* = 0.078). Articles of group C2 were almost all devoted to the evolution of resistance in human parasites: they mainly focused on antibiotic and antiviral drugs (Table 1 and Figure 3). By contrast, articles of group C1 were generally devoted to models of resistance evolution in agricultural settings: they mainly focused on resistance to antihelminthic drugs, fungicides, herbicides, insecticides, or insecticidal proteins (Table 1 and Figure 3). The C1 cluster also included the articles devoted to resistance to antimalarial drugs, but, as indicated above, the association between these articles and the other articles belonging to the group C1 was weak. Groups C1 and C2 were also differentiated by the type of modelling approach: most of the articles of group C1 used population genetics models whereas epidemiological models were dominant in group C2 (Table 1). The first author's discipline also differed between the two groups. Most first authors of C1 articles were biologists whereas there were similar numbers of biologist and medical first authors for C2 articles (Table 1). We confirmed that the ‘isolated articles’ were distributed across the different categories (Table S6).

We further divided the whole network, by at each step splitting the largest remaining group. These additional splits distinguished seven groups within the C1 group. Consequently, the network then had eight groups in total (seven C1 subgroups plus the C2 group). This clustering was statistically validated by the multiresponse permutation procedure (Table S7). The seven C1 subgroups were significantly different (Fisher's exact test, *p*<10^−5^) with respect to the type of pesticide or drug they addressed (Table S8). As expected from the results obtained in our global analysis, the first group that split out from the C1 cluster consisted mostly of articles focusing on resistance to antimalarial drugs. The second group contained only articles focusing on resistance to herbicides. The third and fourth groups were mostly (87.5% and 90%) articles modelling resistance to fungicide and antihelminthic drugs, respectively. The fifth group included all the articles focusing on evolution of resistance in the western corn root worm, *Diabrotica virgifera virgifera*, to *Bt* corn. Finally, 80% of the articles belonging to the sixth and seventh subgroups were devoted to insecticides and insecticidal proteins also included most of the unspecific articles (Figure 3).

### Relationships between citation and authorship network groupings

Note that the A and C groupings described above were obtained independently from each other. Cross-classification of the 187 articles between the authorship and citation groups indicated that the two classifications were however not independent from each other (Table 2, Fisher's exact test, *p*<10^−5^). All articles of the A1 authorship group were classified in the C1 citation group and all except two [14], [15] of the articles of the A2 authorship group belonged to the C2 citation group. Moreover, five of the six ‘isolated articles’ of the citation network were also scored as isolated in the authorship classification (Table 2). The classification of the articles by May and Hassel [14] and Koella and Antia [15] in the C1 group is consistent with our characterization of this group (Table 1) as these articles dealt with pesticide resistance in agricultural settings and resistance to antimalaria drugs, respectively. They were attributed to the A2 authorship because R. M. May is also co-author of articles dealing with HIV resistance to antiviral drugs [16], [17], and R. Antia is also co-author of articles dealing with antibiotic resistance [18], [19]. Because the classifications based on the citation and the authorship networks were very similar and because author groups A1 and A2 were apparently unconnected, we analysed the information flow between groups using the citation network only.

**Table 2:** Contingency table crossing for the authorship and citation groups.

Authorship Network	Citation Network			*Total*
	C1 Group	C2 Group	Isolated Articles	
A1 Group	45	0	0	*45*
A2 Group	2	13	0	*15*
Small Groups	58	19	1	*78*
Isolated Articles	33	12	4	*49*
*Total*	*138*	*44*	*5*	*187*

The independence of each network structure was significantly rejected (Fisher's exact test, *p*<10^−5^).

### Information flow between citation groups

Overall, 48 of the 857 references were quoted both by articles belonging to the C1 group and articles belonging to the C2 group (Table S9). Thus, articles from group C1 and group C2 shared less than 5.6% of the references (after exclusion of all the references that were cited only once). The citation network analysis assigned 39.5% (19 articles) of the 48 shared references to group C1 and 60.5% (29 articles) to group C2; these shared references made up 3% of the total number of C1 citations and 12.8% of the total number of C2 citations, indicating that articles of the group C1 are more prone to quote C2 references that the reverse. The 48 article cited mostly reported models of the evolution of resistance to fungicides (30%), to antibiotics (27%) and general models of theoretical population biology and genetics (16%). To assess the extent of information flow between C1 and C2 groups, we compared the five most frequently quoted references and journals of each group. The results were particularly striking: none of the five most cited references and journals of one group appeared in the top five of the other group (Table 3). Indeed, none of the most frequently quoted references of group C1 was quoted by group C2. Even the keystone article by Comins [20], which was quoted 35 times by group C1, did not appear among the references of the other group. By contrast, the most frequently quoted references of group C2 were in some cases quoted by group C1. For instance, the keystone book by Anderson and May [21], which was cited 18 times by group C2, was also cited four times by group C1 (Table 3). The five top journals cited by group C1 were mainly specialized in entomology. The only journal that was also cited by group C2 was *Phytopathology.* The five top journals cited in the group C2 included two specialized medical journals, that were almost never cited by articles of the C1 group, and three generalist journals–*Science*, *Nature* and *PNAS*–that were frequently cited by articles of the C1 group (Table 3)

**Table 3:** The five most cited references and journals within the citation groups C1 and C2 and number of citations of these references/journals in each group.

Citation Group	Most Cited References	Quotation Number	
		C1	C2
C1	Comins *J. theor. Biol.* (1977) **64**, 177–197	35	0
	Tabashnik et al. *Env. Entomol.* (1982) **11**, 1137–1144	31	0
	Georghiou and Taylor *J. Econ. Entomol* . (1977) **70**, 319–323	26	0
	Roush and McKenzie *Annu. Rev. Entomol.* (1987) **32**, 361–380	23	0
	Gould. *Annu. Rev. Entomol.* (1998) **43**, 701–726	22	0
C2	Anderson and May (1991) *Oxford University Press*	4	18
	Bonhoeffer et al. *Proc. Natl. Acad. Sci. USA* (1997) **94,** 12106–12111	1	12
	Blower et al. *Science* (1996) **273,** 497–500	1	11
	Levin et al. *Clinic. Inf. Dis.* (1997) **24**, S9–S16	0	10
	Wei et al. *Nature* (1995) **373,** 117–122	0	10
	**Most Cited Journals**		
C1	*Journal of Economic Entomology*	614	0
	*Environmental Entomology*	211	0
	*Annual Review of Entomology*	119	0
	*Phytopathology*	108	10
	*Pesticide Science/Pest Management Science*	99	0
C2	*Science*	84	97
	*Proceedings of the National Academy of Sciences of the USA*	74	92
	*Antimicrobial Agents Chemotherapy*	0	79
	*Nature*	53	65
	*New England Journal of Medecine*	2	62

An alternative method for assessing the information flow between citation groups is to examine the articles of one group that cite references assigned to the other group. Only 28 of the articles of the database did so. The 28 articles included half of all articles in our database dealing with antimalarial drug resistance, 33% of the articles dealing with fungicide resistance evolution and 29% of the articles classified as ‘unspecified’ (Table S10). Among the 28 articles: (i) 21 articles, belonging to the group C1, cited at least one reference assigned to the group C2. One of these articles, that by Koella and Antia [15] cited the largest number of references belonging to the C2 group (6 citations). It is an epidemiological model describing the evolution of drug resistance in malaria parasite populations. Note that this article was one of the two C1 articles classified in the A2 authorship group because of R. Antia's interest in antibiotic resistance, which indicates that collaboration between scientists with different interests leads to research based on a wider literature, (ii) seven articles, belonging to the group C2, cited at least one reference assigned to the group C1. The article of Gubbins et al. [22] cited the largest number of references belonging to C1 group (9 citations); it presents a stochastic model of fungicide resistance evolution that quotes six models of antibiotic resistance and three general models from population biology.

## Discussion

The absence of interconnection between scientists modelling the evolution of resistance has already been described [23], [3]. Peck [23] titled his article “*Antibiotic and insecticide resistance modeling—is it time to start talking?*”, illustrating the lack of interdisciplinary work in the field. It was indeed time to talk and there was much to be gained by cross-fertilization between the disciplines. Unfortunately, the article of Peck [23] was to a large extent ignored until 2004 and has not been cited more than six times. This illustrates surprisingly limited interest in this key article — note that other articles published in 2001 in the same journal have been cited (by July 2007 and according to the Web of Science) 17.2 times on average.

Although most people working on the evolution of resistance would agree that there is indeed some compartmentalization in the field, a thorough analysis of how the scientific community is structured had never been conducted, and the factors structuring it had never been identified. By contrasting the biology and genetics of insects and bacteria, Peck [23] restricted his analysis to antibiotics and insecticides ignoring models devoted to other pesticides or drugs. More importantly, the goal of Peck's analysis was not to provide any quantitative measure of the isolation between groups of scientists but to alert the scientific community. He assumed that the community of scientists modelling resistance was significantly structured according to their interest in pesticides or drugs–in his case insecticides and antibiotics. By contrast, our aim was to test for the structure of the community using network analysis and including all major classes of drugs and pesticides. Using a database of 187 articles modelling the evolution of resistance to all the various classes of pesticides and drugs, we performed network analyses with no *a priori* knowledge of view about the factors structuring the community.

Both authorship and citation networks identified two major scientific groups working in parallel in the field of resistance evolution modelling. One group is interested in resistance evolution in agricultural settings (i.e., crop or stock pests or causal agents of disease). It mainly uses a population genetics approach to model the evolution of resistance to insecticidal proteins, insecticides, herbicides, antihelminthic drugs or miticides. By contrast, the other group is interested in resistance evolution in human parasites and predominantly uses epidemiological models to study the evolution of resistance to antibiotic and antiviral drugs. Moreover, our analyses suggest that there is a small scientific group focusing on resistance to antimalarial drugs, and which is only weakly connected to the two major groups. Our analysis of cited references provides strong evidence that each of the two large communities establishes its research in a different set of literature and has its own keystone references. This is well illustrated by the very low percentage (less than 5.6%) of cited references shared by the two communities. The division is even clearer in the structure of collaborations between authors. We identified two large collaborative groups of scientists–one that explores resistance of human parasites and the other that works on insecticide resistance in insect crop pests–that have been coexisting without ever coming together to produce common publications.

Although robust and reliable, this result needs to be qualified. Indeed, there is information flow between the two citation groups although it is low volume. Some of the few references that are cross cited are general developments in the field of ecology and genetics, and this finding indicates that some theoretical literature is shared between citation groups. The articles of one group citing references of the other group, included 50% of all articles concerning antimalarial drug resistance models and 33% of the all those addressing fungicide resistance models. The two articles citing the largest number of references from the other group also focused on fungicide [22] and antimalarial drugs [15]. This is consistent with the type of modelling approach used for work on antimalarial drugs and fungicides. For antimalarial drugs resistance models, epidemiological approaches, typical of human parasite modelling, were used as frequently as population genetics approaches that are typical of agricultural pest modelling [24]. Fungicide models used epidemiological approaches leading the authors to cite literature of the C2 group. In these contexts, the modelling approach compartment might be preferred by the authors because the variable of interest is likely to be symptoms as perceived on the whole plant rather than the cryptic presence of individuals which are not countable. Indeed, the division of this scientific community into two major groups–one affiliated to agriculture and the other to medicine– is not perfectly correlated with the kind of modelling approach used: about 24% of models of C1 group are not population genetics models whereas 4.5% of the articles belonging to C2 group presented population genetics models. This would be expected to generate the need to cross cite references which is clearly not the case.

The compartmentalization between the two groups appears asymmetrical: modelling approaches, geographic origins and disciplines of the first authors are much more diverse for the community of researchers working in medicine than in agriculture (Table 1). In addition, the most cited journals quoted by the authorship group working on human parasite resistance are generalist journals and are also cited by the other group. The reverse is does not apply: the group working on agricultural pest resistance cites specialised (entomology or phytopathology) journals that are not cited by the other group. For these reasons of ‘generalism’, ‘diversity’, and to a lesser degree ‘youth’, we believe that researchers working on the evolution of resistance in the medical sciences may be both more responsive to progress in other scientific fields and better disposed to multidisciplinary research.

The division of research on the evolution of resistance into two communities has, as stated above, already been reported [23], [3]. Hastings [3] suggested that the modelling approach–epidemiology versus population genetics–and the reproduction mode of the organism under study structured the community: epidemiology models being used for asexual species (virus, bacteria, fungi) whereas population genetics models apply to sexually reproducing pathogens and pests. Peck [23] asserted that “*the lack of interdisciplinary work in resistance modelling seems to be that bacterial genetics differ substantially from the genetics of diploid organisms such as insects and mites*”. Our analysis of a large panel of articles suggests that the modelling approach is not the factor that best structured the community. This was clearly illustrated by the literature for antimalarial drugs and fungicides, and by the numerous population genetics models developed to analyse evolutionary outcomes in bacteria [18], [25]–[27]. Moreover, explanations based on differences in reproductive modes or genetics are similarly not entirely satisfactory because large variations in recombination rates in insects (e.g. in aphids [28] as in bacteria [29]), and because the models of population genetics of haploid and diploid organisms are very similar. For instance, the Wright-Fisher and the coalescent models are nearly the same for both haploids and diploids [30].

Finally, contrary to previous suggestions by Hastings [3] and Peck [23], we think that the two separate groups have developed a the result of the more traditional division between research in agriculture (and related sciences) and medical sciences. The coexistence of two communities in the field of resistance evolution modelling–one specialized on agricultural pests or diseases, the other on human parasites–may not have initially arisen from the need to use different modelling approaches because of biological differences in target organisms. Moreover, even if this were the case, the methodology used for modelling should not, *per se*, preclude cross citation and scientific exchange. Given the almost complete absence of citation of the keystone articles of one community by the other community, we suggest that the division corresponds to the independent development of scientific groups around different leaders. Our view is that the article by Comins [20] may have given rise to a lineage of population genetics models on insect pests whereas the book by Anderson and May [21] may have initiated the proliferation of epidemiological models on human diseases. Merging of the two lineages may have been inhibited by the applied nature of research on the evolution of resistance: modelling the evolution of resistance evolution is driven by practical problems encountered by farmers or medical practitioners. Therefore, scientific exchanges may have occurred preferentially between scientists working with the same practical issues, and addressing the same audience. We also think that the current compartmentalization is due to the absence, when this scientific field first emerged, of major contributions by founders presenting general models of resistance evolution.

We fear that this historical, field-oriented division impedes the progress of research at a time when the development of new pesticides and drugs is a growing problem. Possibly, the subdivision into two communities has been beneficial by favouring the emergence of different models over mimicking those already developed in the other community. Hence, although the community is clearly divided into two groups, we now need to investigate whether or not they arrive at similar conclusions and management solutions. These issues will be addressed in a subsequent paper.

## Materials and Methods

### Construction of the database

We established a database of articles presenting models (mathematical models or computer simulations) of the evolution of resistance to the most common classes of pesticides–insecticides, fungicides, herbicides, miticides and insecticidal proteins such as *Bacillus* toxins–and drugs–antibiotic, antiviral, antimalarial and antihelmintic drugs. We used a three-step process to select relevant articles without using any subjective *a priori* knowledge of the relevant literature. First, we selected in the Web of Science (1992–2006) database the four most cited articles concerning models (mathematical models or computer simulations) to study the evolution of resistance to each of the six following pesticides and drugs: insecticides, herbicides, fungicides, insecticidal proteins and antibiotic and antiviral drugs. We used the search formula TS = (model* AND resistan* AND X), with X being one of the six pesticides or drugs under consideration. The most cited articles were then checked to verify their relevance. This resulted in a kernel of 24 ‘core articles’. The second step involved a search in the CABs 1973–2006, Current Contents 1998–2006 and Medline 1950–2006. The aim was to establish, using a trial and error method, a single search formula detecting the smallest set of articles that included at least all the 24 ‘core articles’. The final “formula” used–on September 1st 2006–for the search in the three bibliographic databases is given in Table S11. The third step was to reduce the dataset by removing all irrelevant articles: the summary and keywords of each article were carefully and independently read by two of us to verify that the article dealt with a mathematical model or a computer simulation of the temporal evolution of resistance in response to selective pressures induced by a pesticide or a drug. The final database consisted of a total of 187 articles (see the results section; the references of these articles are given in Table S1).

For each of the selected articles, a reading grid was completed with details of the type of drug or pesticide (insecticides, fungicides, herbicides, miticides, insecticidal proteins, and antibiotic, antiviral, antimalarial and antihelmintic drugs), the kind of target organism (farm pest and disease versus human parasite), the first author affiliations (geographical location of work and scientific discipline as indicated by the institution department). Each article was also classified according to the modelling approach used, according to Levin [27], [31]: the population genetics approach considers the change in the frequencies of resistant and sensitive individuals as function of pesticide or drug use; whereas the epidemiological approach refers to the compartment model tradition of mathematical epidemiology of parasites as described by Anderson and May [21]. Models that could not be assigned to the “population genetics model” nor to the “epidemiological model” were classified as “other”.

### Network construction

All author names and all cited references in each article of the database were recorded. These data were used to build two bipartite undirected networks (R igraph package, *graph.adjacency* function [32], [33]). The edges of the first network linked articles to their authors. This network, named the authorship network, had 508 nodes representing 187 articles connected with 321 authors. The edges of the second network linked articles to their bibliographic references. This network, named the citation network, had 4,387 nodes representing 187 articles connected with 4,154 cited references. The aim was to group articles according to their similarity in authorship or citations, so we removed all the authors who had published only once and all the references cited only once. Articles which were no longer linked to any author or any reference were removed from the networks and were classified as “isolated articles”. This led to a simplified authorship network of 138 articles connected with 87 authors and a simplified citation network of 182 articles connected with 857 citations. These networks had 260 and 2,943 edges, respectively.

### Detection of connected components and clusters

We first identified the connected components of the two networks and measured their sizes using the *clusters* function of the R igraph package [32], [33]. The citation network was found to be fully connected, so the clustering algorithm proposed by Girvan and Newman [13] was used to analyse its structure. This divisive algorithm selects the edges of the network to be cut based on their ‘edge betweenness’, a generalization of the centrality betweenness, originally defined for graph vertices [34], [35]. Edge betweenness is (roughly) equal to the number of shortest paths linking all pairs of vertices going through an edge. It was calculated using the *edge.betweenness* function of the R igraph package [32], [33]. As detailed by Girvan and Newman [13], if a network contains clusters that are loosely connected by few edges, the edges connecting these clusters have a high betweenness because all shortest paths between vertices of different clusters must pass through them. At a given stage, edges with the highest betweenness were therefore removed from the citation network and the betweenness for all the remaining edges was recalculated. This sequence was repeated until separation of clusters. Each cluster was then split in its turn, starting with the largest. The algorithm was run until no edge remained. The nested hierarchy of clusters was converted into a tree format using the *as.phylo.formula* function of the R ape package [33], [36], and is represented as an unrooted radial tree with TreeView [37].

The clustering algorithm was originally designed for unipartite networks [13], so we also applied it to the unipartite projection of the citation network on articles. This projection yielded to a network having 182 nodes representing the 182 articles of the citation network. Two articles were connected with each other if they shared at least one reference. A total of 3,106 edges linked together the articles. We checked that the major divisions of this unipartite network were the same as those of the bipartite citation network.

Finally, we constructed the network with the 187 database articles as vertices and in which two articles were linked together if one cited the other. We found that sixteen articles were not cited by any other article of the database and did not cite any other article. Thus, they were not linked to any other article in the network. They were removed and classified as “isolated” articles. This led to a simplified, fully connected, unipartite network with 171 nodes and 590 edges, which was called the unipartite co-citation network. The information contained in this network was complementary to that of the bipartite citation network. Indeed, in the bipartite citation network, the common references through which the articles were linked were of various types: they were general reviews or books on resistance evolution, theoretical articles on resistance evolution, or specific studies on the biology of target organisms. By contrast, in the unipartite co-citation network, links corresponded to citations between theoretical studies only because our database was cleared to include only models of resistance evolution. The bipartite citation network therefore provides a general, exhaustive picture of knowledge flow between the 187 database articles, whereas the unipartite co-citation network gives an insight into the flow of theoretical knowledge only. We studied the architecture of the unipartite co-citation network with the clustering algorithm and compared it with that of the bipartite citation network.

### Statistical validation of clusters

The clusters obtained by clustering algorithm analysis with the bipartite citation network were statistically validated by testing whether element similarity–*i.e*. article similarity according to their cited references and reference similarity according to their source articles–was significantly higher within than among clusters. For this, we used the multiresponse permutation procedure (MRPP), a non-parametric method designed for testing differences among *a priori* defined groups [38]. The MRPP statistic *δ* is the weighted within-group mean of the pairwise dissimilarities among their elements. Using the method of Prado et al. [39], dissimilarity was calculated here as a Jaccard distance and group size was taken for group weighting. The permutation algorithm included in the *mrpp* function of the R vegan package [33], [40] calculates the expected statistics E(*δ*) if groups were assembled at random. The within-group chance-corrected agreement (A), defined as 1-*δ*/E(*δ*), has a maximum value of 1 when there is no dissimilarity among elements of any groups. The *p*-value is the probability of obtaining by chance a value of A equal or larger than the observed value.

## Supporting Information

Table S1 References of the 187 articles included in the database
(0.09 MB PDF)

Table S2 Number of articles falling into the various descriptive categories for each group of the authorship network
(0.03 MB PDF)

Table S3 Multiresponse permutation procedure (MRPP) analysis of group dissimilarities showing mean citation distance between articles and mean source articles distance between citations in each citation group
(0.02 MB PDF)

Table S4 Contingency table crossing for citation groups obtained by applying the clustering algorithm to the bipartite citation and to the unipartite article networks
(0.01 MB PDF)

Table S5 Contingency table crossing for citation groups obtained by applying the clustering algorithm to the bipartite citation and to the unipartite co-citation networks
(0.01 MB PDF)

Table S6 Number of articles falling into the various descriptive categories for each group of the citation network
(0.03 MB PDF)

Table S7 Multiresponse permutation procedure (MRPP) analysis of group dissimilarities showing mean citation distance between articles and mean source article distance between citations in each citation group
(0.02 MB PDF)

Table S8 Number of articles focusing on the different types of drug or pesticide for each subgroup of the C1 cluster
(0.02 MB PDF)

Table S9 List of the 48 references cited by articles belonging to the C1 group and articles belonging to the C2 group
(0.02 MB PDF)

Table S10 Characteristics of the 28 articles of one group that cited references of the other group
(0.02 MB PDF)

Table S11 Formulae used to search for relevant articles describing models of the evolution of resistance to pesticides and drugs within the CABs (1973–2006), Current Contents (1998–2006), and Medline (1950–2006) databases
(0.02 MB PDF)
