# Supplementary material for: Structure of the Scientific Community Modelling the Evolution of Resistance
Source: PLoS One. 2007 Dec 5;2(12):e1275. doi: 10.1371/journal.pone.0001275 (PMC2094735; doi:10.1371/journal.pone.0001275)
Supplement: Table S1 — References of the 187 articles included in the database (0.09 MB PDF) [file pone.0001275.s001.pdf]

Table S1. References of the 187 articles included in the database.

1. Alstad, D. N. and D. A. Andow (1995). "Managing the Evolution of Insect Resistance to Transgenic Plants." Science **268**(5219): 1894-1896.
2. Argentine, J. A., J. M. Clark, et al. (1994). "Computer simulation of insecticide resistance management strategies for control of Colorado potato beetle (Coleoptera: Chrysomelidae)." Journal of Agricultural Entomology **11**(2): 137-155.
3. Arpaia, S., K. Chiriatti, et al. (1998). "Predicting the adaptation of Colorado potato beetle (Coleoptera: Chrysomelidae) to transgenic eggplants expressing CryIII toxin: The role of gene dominance, migration, and fitness costs." Journal of Economic Entomology **91**(1): 21-29.
4. Austin, D. J. and R. M. Anderson (1999). "Studies of antibiotic resistance within the patient, hospitals and the community using simple mathematical models." Philos Trans R Soc Lond B Biol Sci **354**(1384): 721-38.
5. Austin, D. J., M. Kakehashi, et al. (1997). "The transmission dynamics of antibiotic-resistant bacteria: the relationship between resistance in commensal organisms and antibiotic consumption." Proc Biol Sci **264**(1388): 1629-38.
6. Austin, D. J., K. G. Kristinsson, et al. (1999). "The relationship between the volume of antimicrobial consumption in human communities and the frequency of resistance." Proc Natl Acad Sci U S A **96**(3): 1152-6.
7. Barnes, E. H. and R. J. Dobson (1990). "Population dynamics of Trichostrongylus colubriformis in sheep: computer model to simulate grazing systems and the evolution of anthelmintic resistance." International Journal for Parasitology **20**(7): 823-831.
8. Barnes, E. H., R. J. Dobson, et al. (1995). "Worm control and anthelmintic resistance: adventures with a model." Parasitology Today **11**(2): 56-63.
9. Benderly, M. and Y. Levy (1988). Modeling the buildup of multigenic resistance to systemic fungicides in a haploid fungal pathogen. Mededelingen van de Faculteit Landbouwwetenschappen, Rijksuniversiteit Gent. **53:** 589-595.
10. Bergstrom, C. T., M. Lo, et al. (2004). "Ecological theory suggests that antimicrobial cycling will not reduce antimicrobial resistance in hospitals." Proceedings of the National Academy of Sciences of the United States of America **101**(36): 13285-13290.
11. Birch, C. P. D. and M. W. Shaw (1997). "When can reduced doses and pesticide mixtures delay the build-up of pesticide resistance? A mathematical model." Journal of Applied Ecology **34**(4): 1032-1042.
12. Blower, S. M., T. C. Porco, et al. (1998). "Predicting and preventing the emergence of antiviral drug resistance in HSV-2." Nat Med **4**(6): 673-8.
13. Bonhoeffer, S., M. Lipsitch, et al. (1997). "Evaluating treatment protocols to prevent antibiotic resistance." Proc Natl Acad Sci U S A **94**(22): 12106-11.
14. Bonhoeffer, S., R. M. May, et al. (1997). "Virus dynamics and drug therapy." Proc Natl Acad Sci U S A **94**(13): 6971-6.
15. Bonhoeffer, S. and M. A. Nowak (1997). "Pre-existence and emergence of drug resistance in HIV-1 infection." Proc Biol Sci **264**(1382): 631-7.
16. Boni, M. F. and M. W. Feldman (2005). "Evolution of antibiotic resistance by human and bacterial niche construction." Evolution Int J Org Evolution **59**(3): 477-91.
17. Caprio, M. A. (1998). "Evaluating resistance management strategies for multiple toxins in the presence of external refuges." Journal of Economic Entomology **91**(5): 1021-1031.
18. Caprio, M. A. (2001). "Source-sink dynamics between transgenic and non-transgenic habitats and their role in the evolution of resistance." J Econ Entomol **94**(3): 698-705.
19. Caprio, M. A. and M. A. Hoy (1994). "Metapopulation dynamics affect resistance development in the predatory mite, Metaseiulus occidentalis (Acari: Phytoseiidae)." Journal of Economic Entomology **87**(3): 525-534.

20. Caprio, M. A. and M. A. Hoy (1995). "Premating isolation in a simulation model generates frequency-dependent selection and alters establishment rates of resistant natural enemies." Journal of Economic Entomology **88**(2): 205-212.
21. Caprio, M. A. and D. M. Suckling (2000). "Simulating the impact of cross resistance between Bt toxins in transformed clover and apples in New Zealand." J Econ Entomol **93**(2): 173-9.
22. Caprio, M. A. and B. E. Tabashnik (1992). "Gene flow accelerates local adaptation among finite populations: simulating the evolution of insecticide resistance." Journal of Economic Entomology **85**(3): 611-620.
23. Carrière, Y. (2003). "Haplodiploidy, sex, and the evolution of pesticide resistance." Journal of Economic Entomology **96**(6): 1626-1640.
24. Carrière, Y., C. Ellers-Kirk, et al. (2001). "Predicting spring moth emergence in the pink bollworm (Lepidoptera: Gelechiidae): implications for managing resistance to transgenic cotton." J Econ Entomol **94**(5): 1012-21.
25. Carrière, Y. and B. E. Tabashnik (2001). "Reversing insect adaptation to transgenic insecticidal plants." Proc Biol Sci **268**(1475): 1475-80.
26. Castillo-Chavez, C. and Z. Feng (1997). "To treat or not to treat: the case of tuberculosis." J Math Biol **35**(6): 629-56.
27. Cavan, G., J. Cussans, et al. (2000). "Modelling different cultivation and herbicide strategies for their effect on herbicide resistance in Alopecurus myosuroides." Weed Research **40**(6): 561-568.
28. Cavan, G., J. Cussans, et al. (2001). "Managing the risks of herbicide resistance in wild oat." Weed Science **49**(2): 236-240.
29. Cerda, H. (2002). "A high-dose refugia management strategy for Bt transgenic crops: spraying the refugia." Antenna (London) **26**(2): 124-126.
30. Chilcutt, C. F. and B. E. Tabashnik (1999). "Simulation of integration of Bacillus thuringiensis and the parasitoid Cotesia plutellae (Hymenoptera: Braconidae) for control of susceptible and resistant diamondback moth (Lepidoptera: Plutellidae)." Environmental Entomology **28**(3): 505-512.
31. Chin, K. M. (1987). "A simple method of selection for fungicide resistance in plant pathogen populations." Phytopathology **77**(5): 666-669.
32. Cohen, T. and M. Murray (2004). "Modeling epidemics of multidrug-resistant M-tuberculosis of heterogeneous fitness." Nature Medicine **10**(10): 1117-1121.
33. Comins, H. N. (1977). "The development of insecticide resistance in the presence of migration." J Theor Biol **64**(1): 177-97.
34. Comins, H. N. (1979). "Analytic methods for the management of pesticide resistance." J Theor Biol **77**(2): 171-88.
35. Comins, H. N. (1986). "Tactics for resistance management using multiple pesticides." Agriculture, Ecosystems and Environment **16**(2): 129-148.
36. Coop, L. B., B. A. Croft, et al. (1994). Geographic information systems and simulation in regional orchard management: pesticide resistance in Hood River valley, Oregon. Acta Horticulturae**:** 383-390.
37. Cornell, S. J., V. S. Isham, et al. (2003). "Spatial parasite transmission, drug resistance, and the spread of rare genes." Proceedings of the National Academy of Sciences of the United States of America **100**(12): 7401-7405.
38. Cross, A. P. and B. Singer (1991). "Modelling the development of resistance of Plasmodium falciparum to anti-malarial drugs." Transactions of the Royal Society of Tropical Medicine and Hygiene **85**(3): 349-355.
39. Crowder, D. W. and D. W. Onstad (2005). "Using a generational time-step model to simulate dynamics of adaptation to transgenic corn and crop rotation by western corn rootworm

(Coleoptera: Chrysomelidae)." Journal of Economic Entomology **98**(2): 518-533.

40. Crowder, D. W., D. W. Onstad, et al. (2005). "Analysis of the dynamics of adaptation to transgenic corn and crop rotation by western corn rootworm (Coleoptera: Chrysomelidae) using a daily time-step model." Journal of Economic Entomology **98**(2): 534-551.

41. Curtis, C. F., N. Hill, et al. (1993). "Are there effective resistance management strategies for vectors of human disease?" Biological Journal of the Linnean Society **48**(1): 3-18.

42. Curtis, C. F. and L. N. Otoo (1986). "A simple model of the build-up of resistance to mixtures of anti-malarial drugs." Transactions of the Royal Society of Tropical Medicine and Hygiene **80**(6): 889-892.

43. D'Agata, E. M. C., G. F. Webb, et al. (2005). "A mathematical model quantifying the impact of antibiotic exposure and other interventions on the endemic prevalence of vancomycin-resistant enterococci." J Infect Dis **192**(11): 2004-11.

44. De Boer, R. J. and C. A. Boucher (1996). "Anti-CD4 therapy for AIDS suggested by mathematical models." Proc Biol Sci **263**(1372): 899-905.

45. De Souza, K., J. Holt, et al. (1995). "Diapause, migration and pyrethroid-resistance dynamics in the cotton bollworm, *Helicoverpa armigera* (Lepidoptera: Noctuidae)." Ecological Entomology **20**(4): 333-342.

46. Diggle, A. J., P. B. Neve, et al. (2003). "Herbicides used in combination can reduce the probability of herbicide resistance in finite weed populations." Weed Research **43**(5): 371-382.

47. Dobson, R. J., D. A. Griffiths, et al. (1987). "A genetic model describing the evolution of levamisole resistance in Trichostrongylus colubriformis, a nematode parasite of sheep." IMA Journal of Mathematics Applied in Medicine and Biology **4**(4): 279-293.

48. Doster, M. A., M. G. Milgroom, et al. (1990). "Quantification of factors influencing potato late blight suppression and selection for metalaxyl resistance in Phytophthora infestans: a simulation approach." Phytopathology **80**(11): 1190-1198.

49. Dugatkin, L. A., M. Perlin, et al. (2005). "Antibiotic resistance and the evolution of group-beneficial traits. II: a metapopulation model." J Theor Biol **236**(4): 392-6.

50. Dye, C. and M. A. Espinal (2001). "Will tuberculosis become resistant to all antibiotics?" Proceedings of the Royal Society of London Series B-Biological Sciences **268**(1462): 45-52.

51. Dye, C. and B. G. Williams (2000). "Criteria for the control of drug-resistant tuberculosis." Proc Natl Acad Sci U S A **97**(14): 8180-5.

52. Echevarria, F. A. M., G. Gettinby, et al. (1993). "Model predictions for anthelmintic resistance amongst Haemonchus contortus populations in southern Brazil." Veterinary Parasitology **47**(3-4): 315-325.

53. Emery, V. C. and P. D. Griffiths (2000). "Prediction of cytomegalovirus load and resistance patterns after antiviral chemotherapy." Proceedings of the National Academy of Sciences of the United States of America **97**(14): 8039-8044.

54. Follett, P. A., F. Gould, et al. (1995). "High-realism model of Colorado potato beetle (Coleoptera: Chrysomelidae) adaptation to permethrin." Environmental Entomology **24**(2): 167-178.

55. Follett, P. A., G. G. Kennedy, et al. (1993). "REPO: a stimulation model that explores Colorado potato beetle (Coleoptera: Chrysomelidae) adaptation to insecticides." Environmental Entomology **22**(2): 283-296.

56. Garber, A. M. (1987). "Antibiotic exposure and resistance in mixed bacterial populations." Theor Popul Biol **32**(3): 326-46.

57. Gardner, S. N., J. Gressel, et al. (1998). "A revolving dose strategy to delay the evolution of both quantitative vs major monogene resistances to pesticides and drugs." International Journal of Pest Management **44**(3): 161-180.

58. Gatton, M. L., W. Hogarth, et al. (2001). "Time of treatment influences the appearance of

drug-resistant parasites in Plasmodium falciparum infections." Parasitology **123**(6): 537-546.
59. Gazzoni, D. L. (1998). "Modeling insect resistance to insecticides using velvetbean caterpillar (Anticarsia gemmatalis) as an example." Pesticide Science **53**(2): 109-122.
60. Georghiou, G. P. and C. E. Taylor (1977). "Genetic and biological influences in the evolution of insecticide resistance." J Econ Entomol **70**(3): 319-23.
61. Gershengorn, H. B. and S. M. Blower (2000). "Impact of antivirals and emergence of drug resistance: HSV-2 epidemic control." AIDS Patient Care STDS **14**(3): 133-42.
62. Gettinby, G., R. M. Newson, et al. (1988). "A simulation model for genetic resistance to acaricides in the African brown ear tick, Rhipicephalus appendiculatus (Acarina: Ixodidae)." Preventive Veterinary Medicine **6**(3): 183-197.
63. Gettinby, G., A. Soutar, et al. (1989). "Anthelmintic resistance and the control of ovine ostertagiasis: a drug action model for genetic selection." International Journal for Parasitology **19**(4): 369-376.
64. Gorddard, R. J., D. J. Pannell, et al. (1995). "An optimal control model for integrated weed management under herbicide resistance." Australian Journal of Agricultural Economics **39**(1): 71-87.
65. Gorddard, R. J., D. J. Pannell, et al. (1996). "Economic evaluation of strategies for management of herbicide resistance." Agricultural Systems **51**(3): 281-298.
66. Gould, F. (1986). "Simulation models for predicting durability of insect-resistant germ plasm: a deterministic diploid, two-locus model." Environmental Entomology **15**(1): 1-10.
67. Gould, F. (1994). Potential and problems with high-dose strategies for pesticidal engineered crops. Biocontrol Science and Technology. **4:** 451-461.
68. Gressel, J. and L. A. Segel (1990). "Modelling the effectiveness of herbicide resistance and mixtures as strategies to delay or preclude resistance." Weed Technology **4**(1): 186-198.
69. Gressel, J., L. A. Segel, et al. (1996). "Managing the delay of evolution of herbicide resistance in parasitic weeds." International Journal of Pest Management **42**(2): 113-129.
70. Groeters, F. R. and B. E. Tabashnik (2000). "Roles of selection intensity, major genes, and minor genes in evolution of insecticide resistance." J Econ Entomol **93**(6): 1580-7.
71. Gubbins, S. and C. A. Gilligan (1999). "Invasion thresholds for fungicide resistance: deterministic and stochastic analyses." Proceedings of the Royal Society of London Series B-Biological Sciences **266**(1437): 2539-2549.
72. Gutierrez, A. P., U. Regev, et al. (1979). "An economic optimization model of pesticide resistance: alfalfa and Egyptian alfalfa weevil - an example." Environmental Entomology **8**(1): 101-107.
73. Hall, R. J., S. Gubbins, et al. (2004). "Invasion of drug and pesticide resistance is determined by a trade-off between treatment efficacy and relative fitness." Bull Math Biol **66**(4): 825-40.
74. Hanson, D. E., D. A. Ball, et al. (2002). "Herbicide resistance in jointed goatgrass (Aegilops cylindrica): Simulated responses to agronomic practices." Weed Technology **16**(1): 156-163.
75. Hastings, I. M. (1997). "A model for the origins and spread of drug-resistant malaria." Parasitology **115**(2): 133-141.
76. Hastings, I. M. and M. J. Mackinnon (1998). "The emergence of drug-resistant malaria." Parasitology **117**(5): 411-417.
77. Hastings, I. M., W. M. Watkins, et al. (2002). "The evolution of drug-resistant malaria: the role of drug elimination half-life." Philos Trans R Soc Lond B Biol Sci **357**(1420): 505-19.
78. Heimpel, G. E., C. Neuhauser, et al. (2005). "Natural enemies and the evolution of resistance to transgenic insecticidal crops by pest insects: The role of egg mortality." Environmental Entomology **34**(3): 512-526.
79. Hillier, J. G. and A. N. E. Birch (2002). "A bi-trophic mathematical model for pest adaptation to a resistant crop." Journal of Theoretical Biology **215**(3): 305-319.
80. Hillier, J. G. and A. N. E. Birch (2002). "Travelling waves of resistance in a bi-trophic pest

adaptation model." Journal of Theoretical Biology **219**(4): 507-519.

81. Hoshen, M. B., W. D. Stein, et al. (2002). "Mathematical modelling of malaria chemotherapy: combining artesunate and mefloquine." Parasitology **124**(1): 9-15.

82. Howard, D. H. (2004). "Resistance-induced antibiotic substitution." Health Econ **13**(6): 585-95.

83. Huang, Y., S. L. Rosenkranz, et al. (2003). "Modeling HIV dynamics and antiviral response with consideration of time-varying drug exposures, adherence and phenotypic sensitivity." Math Biosci **184**(2): 165-86.

84. Ives, A. R. and D. A. Andow (2002). "Evolution of resistance to Bt crops: directional selection in structured environments." Ecology Letters **5**(6): 792-801.

85. Jaffe, K., S. Issa, et al. (1997). "Dynamics of the emergence of genetic resistance to biocides among asexual and sexual organisms." J Theor Biol **188**(3): 289-99.

86. Janssen, M. A. and W. J. Martens (1997). "Modeling malaria as a complex adaptive system." Artif Life **3**(3): 213-36.

87. Jasieniuk, M., A. L. Brule-Babel, et al. (1996). "The evolution and genetics of herbicide resistance in weeds." Weed Science **44**(1): 176-193.

88. Josepovits, G. (1989). "A model for evaluating factors affecting the development of insensitivity to fungicides." Crop Protection **8**(2): 106-113.

89. Kawaguchi, I., A. Sasaki, et al. (2004). "Combining zooprophylaxis and insecticide spraying: a malaria-control strategy limiting the development of insecticide resistance in vector mosquitoes." Proc Biol Sci **271**(1536): 301-9.

90. Kelman, E., R. S. Levy, et al. (2001). "Optimization of solutions for the one plant protection problem." Acta Biotheor **49**(1): 61-71.

91. Kirschner, D. E. and G. F. Webb (1997). "Understanding drug resistance for monotherapy treatment of HIV infection." Bulletin of Mathematical Biology **59**(4): 763-785.

92. Knipling, E. F. and W. Klassen (1984). "Influence of insecticide use patterns on the development of resistance to insecticides-a theoretical study." Southwestern Entomologist **9**(3): 351-368.

93. Koella, J. C. and R. Antia (2003). "Epidemiological models for the spread of anti-malarial resistance." Malaria Journal **2**(3): (19 February 2003).

94. Kranthi, K. R. and N. R. Kranthi (2004). "Modelling adaptabiity of cotton bollworm, Helicoverpa armigera (Hubner) to Bt-cotton in India." Current Science **87**(8): 1096-1107.

95. Laxminarayan, R. (2004). "ACT now or later? Economics of malaria resistance." American Journal of Tropical Medicine and Hygiene **71**(2 supplement): 187-195.

96. Leathwick, D. M., A. Vlassoff, et al. (1995). "A model for nematodiasis in New Zealand lambs: the effect of drenching regime and grazing management on the development of anthelmintic resistance." International Journal for Parasitology **25**(12): 1479-1490.

97. Lenormand, T. and M. Raymond (1998). "Resistance management: the stable zone strategy." Proceedings of the Royal Society of London Series B-Biological Sciences **265**(1409): 1985-1990.

98. Levin, B. R. (2001). "Minimizing potential resistance: A population dynamics view." Clinical Infectious Diseases **33**: S161-S169.

99. Levin, B. R., M. Lipsitch, et al. (1997). "The population genetics of antibiotic resistance." Clin Infect Dis **24 Suppl 1**: S9-16.

100. Levy, Y., Y. Cohen, et al. (1991). "Disease development and buildup of resistance to oxadixyl in potato crops inoculated with Phytophthora infestans as affected by oxadixyl and oxadixyl mixtures: experimental and simulation studies." Journal of Phytopathology **132**(3): 219-229.

101. Levy, Y., R. Levi, et al. (1983). "Buildup of a pathogen subpopulation resistant to a systemic fungicide under various control strategies: a flexible simulation model." Phytopathology

73(11): 1475-1480.

102. Levy, Y. and R. S. Levy (1986). "Control strategies using systemic fungicides for limiting disease development and resistance buildup: practical implications of a simulation model." Phytoparasitica **14**(4): 303-312.

103. Li, R. C., D. E. Nix, et al. (1994). "Pharmacodynamic modeling of bacterial kinetics: beta-lactam antibiotics against Escherichia coli." J Pharm Sci **83**(7): 970-5.

104. Lipsitch, M. (2001). "Measuring and interpreting associations between antibiotic use and penicillin resistance in Streptococcus pneumoniae." Clinical Infectious Diseases **32**(7): 1044-1054.

105. Lipsitch, M., C. T. Bergstrom, et al. (2000). "The epidemiology of antibiotic resistance in hospitals: paradoxes and prescriptions." Proc Natl Acad Sci U S A **97**(4): 1938-43.

106. Lipsitch, M. and B. R. Levin (1997). "The population dynamics of antimicrobial chemotherapy." Antimicrob Agents Chemother **41**(2): 363-73.

107. Livingston, M. J., G. A. Carlson, et al. (2002). "Use of mathematical models to estimate characteristics of pyrethroid resistance in tobacco budworm and bollworm (Lepidoptera: Noctuidae) field populations." Journal of Economic Entomology **95**(5): 1008-1017.

108. Longstaff, B. C. (1988). "Temperature manipulation and the management of insecticide resistance in stored grain pests: a stimulation study for the rice weevil, Sitophilus oryzae." Ecological Modelling **43**(3-4): 303-313.

109. Mackinnon, M. J. and I. M. Hastings (1998). "The evolution of multiple drug resistance in malaria parasites." Transactions of the Royal Society of Tropical Medicine and Hygiene **92**(2): 188-195.

110. Madden, A. D. (1995). "An assessment, using a modelling approach, of inbreeding as a possible cause of reduced competitiveness in triazine-resistant weeds." Weed Research (Oxford) **35**(4): 289-294.

111. Madden, A. D., J. Holt, et al. (1995). "The role of uncultivated hosts in the spread of pyrethroid resistance in Helicoverpa armigera populations in Andhra Pradesh, India: a simulation approach." Ecological Modelling **82**(1): 61-74.

112. Madsen, K. H., B. E. Valverde, et al. (2002). "Risk assessment of herbicide-resistant crops: A Latin American perspective using rice (Oryza sativa) as a model." Weed Technology **16**(1): 215-223.

113. Magee, J. T. (2005). "The resistance ratchet: theoretical implications of cyclic selection pressure." J Antimicrob Chemother **56**(2): 427-30.

114. Mallet, J. and R. Luttrell (1991). "A model of insecticidal control failure: the example of Heliothis virescens on cotton." Southwestern Entomologist(No. 15, Supplement): 201-212.

115. Mallet, J. and P. Porter (1992). "Preventing Insect Adaptation to Insect-Resistant Crops - Are Seed Mixtures or Refugia the Best Strategy." Proceedings of the Royal Society of London Series B-Biological Sciences **250**(1328): 165-169.

116. Mangel, M. and R. E. Plant (1983). "Multiseasonal management of an agricultural pest. I: development of the theory." Ecological Modelling **20**(1): 1-19.

117. Mani, G. S. (1989). "Evolution of resistance with sequential application of insecticides in time and space." Proc R Soc Lond B Biol Sci **238**(1292): 245-76.

118. Mason, G. A., B. E. Tabashnik, et al. (1989). "Effects of biological and operational factors on evolution of insecticide resistance in Liriomyza (Diptera: Agromyzidae)." Journal of Economic Entomology **82**(2): 369-373.

119. Massad, E., S. Lundberg, et al. (1993). "Modeling and simulating the evolution of resistance against antibiotics." Int J Biomed Comput **33**(1): 65-81.

120. Maxwell, B. D. (1992). Weed thresholds: the space component and considerations for herbicide resistance. Weed Technology. **6:** 205-212.

121. Maxwell, B. D., M. L. Roush, et al. (1990). "Predicting the evolution and dynamics of

herbicide resistance in weed populations." Weed Technology **4**(1): 2-13.

122. May, R. M. and M. P. Hassell (1988). "Population dynamics and biological control." Philosophical Transactions of the Royal Society of London, B **318**: 129-169.

123. Medvinsky, A. B., A. Y. Morozov, et al. (2004). "Modeling the invasion of recessive Bt-resistant insects: an impact on transgenic plants." J Theor Biol **231**(1): 121-7.

124. Michael, E., M. N. Malecela-Lazaro, et al. (2004). "Mathematical modelling and the control of lymphatic filariasis." Lancet Infectious Diseases **4**(4): 223-234.

125. Milgroom, M. G. (1990). "A stochastic model for the initial occurrence and development of fungicide resistance in plant pathogen populations." Phytopathology **80**(4): 410-416.

126. Milgroom, M. G. and W. E. Fry (1988). "A simulation analysis of the epidemiological principles for fungicide resistance management in pathogen populations." Phytopathology **78**(5): 565-570.

127. Mitchell, P. D. and D. W. Onstad (2005). "Effect of extended diapause on evolution of resistance to transgenic Bacillus thuringiensis corn by northern corn rootworm (Coleoptera: Chrysomelidae)." Journal of Economic Entomology **98**(6): 2220-2234.

128. Muggleton, J. (1986). "Selection for malathion resistance in Oryzaephilus surinamensis (L.) (Coleoptera: Silvanidae): fitness values of resistant and susceptible phenotypes and their inclusion in a general model describing the spread of resistance." Bulletin of Entomological Research **76**(3): 469-480.

129. Munro, A. (1997). "Economics and biological evolution." Environmental and Resource Economics **9**(4): 429-449.

130. Neve, P. B., A. J. Diggle, et al. (2003). "Simulating evolution of glyphosate resistance in Lolium rigidum I: population biology of a rare resistance trait." Weed Research **43**(6): 404-417.

131. Neve, P. B., A. J. Diggle, et al. (2003). "Simulating evolution of glyphosate resistance in Lolium rigidum II: past, present and future glyphosate use in Australian cropping." Weed Research **43**(6): 418-427.

132. Nibouche, S., P. Martin, et al. (2003). "A modelling approach of the sustainability of Bt Cotton grown by small farmers in West Africa." Resistant Pest Management Newsletter **13**(1): 55-58.

133. Nowak, M. A., S. Bonhoeffer, et al. (1997). "Anti-viral drug treatment: dynamics of resistance in free virus and infected cell populations." J Theor Biol **184**(2): 203-17.

134. Onstad, D. W. and F. Gould (1998). "Do dynamics of crop maturation and herbivorous insect life cycle influence the risk of adaptation to toxins in transgenic host plants?" Environmental Entomology **27**(3): 517-522.

135. Onstad, D. W. and F. Gould (1998). "Modeling the dynamics of adaptation to transgenic maize by European corn borer (Lepidoptera: Pyralidae)." Journal of Economic Entomology **91**(3): 585-593.

136. Onstad, D. W. and C. A. Guse (1999). "Economic analysis of transgenic maize and nontransgenic refuges for managing European corn borer (Lepidoptera: Pyralidae)." Journal of Economic Entomology **92**(6): 1256-1265.

137. Onstad, D. W., C. A. Guse, et al. (2002). "Modeling the development of resistance by stalk-boring lepidopteran insects (Crambidae) in areas with transgenic corn and frequent insecticide use." J Econ Entomol **95**(5): 1033-43.

138. Onstad, D. W., C. A. Guse, et al. (2001). "Modeling the dynamics of adaptation to transgenic corn by western corn rootworm (Coleoptera: Chrysomelidae)." Journal of Economic Entomology **94**(2): 529-540.

139. Parnell, S., C. A. Gilligan, et al. (2005). "Small-scale fungicide spray heterogeneity and the coexistence of resistant and sensitive pathogen strains." Phytopathology **95**(6): 632-639.

140. Peck, S. L. and S. P. Ellner (1997). "The effect of economic thresholds and life-history

parameters on the evolution of pesticide resistance in a regional setting." American Naturalist **149**(1): 43-63.

141. Peck, S. L., F. Gould, et al. (1999). "Spread of resistance in spatially extended regions of transgenic cotton: Implications for management of Heliothis virescens (Lepidoptera: Noctuidae)." Journal of Economic Entomology **92**(1): 1-16.

142. Pittendrigh, B. R. and P. J. Gaffney (2001). "Pesticide resistance: can we make it a renewable resource?" J Theor Biol **211**(4): 365-75.

143. Pittendrigh, B. R., P. J. Gaffney, et al. (2004). ""Active" refuges can inhibit the evolution of resistance in insects towards transgenic insect-resistant plants." J Theor Biol **231**(4): 461-74.

144. Pittendrigh, B. R., P. J. Gaffney, et al. (2000). "Deterministic modeling of negative cross-resistance strategies for use in transgenic host-plant resistance." J Theor Biol **204**(1): 135-50.

145. Plapp, F. W., C. R. Browning, et al. (1979). "Analysis of rate of development of insecticide resistance based on simulation of a genetic model." Environmental Entomology **8**(3): 494-500.

146. Rainbolt, C. R., D. C. Thill, et al. (2004). "Herbicide-resistant grass weed development in imidazolinone-resistant wheat: Weed biology and herbicide rotation." Weed Technology **18**(3): 860-868.

147. Reluga, T. C. (2005). "Simple models of antibiotic cycling." Math Med Biol **22**(2): 187-208.

148. Richter, O., P. Zwerger, et al. (2002). "Modelling spatio-temporal dynamics of herbicide resistance." Weed Research **42**(1): 52-64.

149. Roberts, D. E. and R. M. Ribeiro (2001). "Comparison of different treatment regimens for the emergence of new resistance under therapy." JAIDS, Journal of Acquired Immune Deficiency Syndromes **27**(4): 331-335.

150. Rosenheim, J. A. and B. E. Tabashnik (1990). "Evolution of pesticide resistance: interactions between generation time and genetic, ecological, and operational factors." Journal of Economic Entomology **83**(4): 1184-1193.

151. Roush, R. T. (1994). Managing pests and their resistance to Bacillus thuringiensis: can transgenic crops be better than sprays? Biocontrol Science and Technology. **4:** 501-516.

152. Roush, R. T. (1998). "Two-toxin strategies for management of insecticidal transgenic crops: can pyramiding succeed where pesticide mixtures have not?" Philosophical Transactions of the Royal Society of London Series B-Biological Sciences **353**(1376): 1777-1786.

153. Ruppel, R. F. (1983). "Some observations on insecticide resistance." Great Lakes Entomologist **16**(4): 101-107.

154. Samore, M. H., M. Lipsitch, et al. (2006). "Mechanisms by which antibiotics promote dissemination of resistant pneumococci in human populations." American Journal of Epidemiology **163**(2): 160-170.

155. Sebille, V., S. Chevret, et al. (1997). "Modeling the spread of resistant nosocomial pathogens in an intensive-care unit." Infect Control Hosp Epidemiol **18**(2): 84-92.

156. Shaw, M. W. (1989). "Independent action of fungicides and its consequences for strategies to retard the evolution of fungicide resistance." Crop Protection **8**(6): 405-411.

157. Shaw, M. W. (1989). "A model of the evolution of polygenically controlled fungicide resistance." Plant Pathology **38**(1): 44-55.

158. Shaw, M. W. (1993). "Theoretical analysis of the effect of interacting activities on the rate of selection for combined resistance to fungicide mixtures." Crop Protection **12**(2): 120-126.

159. Shaw, M. W. (2000). "Models of the effects of dose heterogeneity and escape on selection pressure for pesticide resistance." Phytopathology **90**(4): 333-339.

160. Sisterson, M. S., L. Antilla, et al. (2004). "Effects of insect population size on evolution of resistance to transgenic crops." J Econ Entomol **97**(4): 1413-24.

161. Sisterson, M. S., Y. Carrière, et al. (2005). "Evolution of resistance to transgenic crops: Interactions between insect movement and field distribution." Journal of Economic

Entomology **98**(6): 1751-1762.

162. Smith, G. (1990). "A mathematical model for the evolution of anthelmintic resistance in a direct life cycle nematode parasite." International Journal for Parasitology **20**(7): 913-921.

163. Smith, G., B. T. Grenfell, et al. (1999). Anthelmintic resistance revisited: under-dosing, chemoprophylactic strategies, and mating probabilities. International Journal for Parasitology. **29:** 77-91.

164. Stewart, F. M., R. Antia, et al. (1998). "The population genetics of antibiotic resistance. II: Analytic theory for sustained populations of bacteria in a community of hosts." Theor Popul Biol **53**(2): 152-65.

165. Stilianakis, N. I., C. A. Boucher, et al. (1997). "Clinical data sets to human immunodeficiency virus type 1 reverse transcriptase-resistant mutants explained by a mathematical model." Journal of Virology **71**(1): 161-168.

166. Stilianakis, N. I., A. S. Perelson, et al. (1998). "Emergence of drug resistance during an influenza epidemic: insights from a mathematical model." J Infect Dis **177**(4): 863-73.

167. Storer, N. P. (2003). "A spatially explicit model simulating western corn rootworm (Coleoptera: Chrysomelidae) adaptation to insect-resistant maize." J Econ Entomol **96**(5): 1530-47.

168. Storer, N. P., S. L. Peck, et al. (2003). "Sensitivity analysis of a spatially-explicit stochastic simulation model of the evolution of resistance in Helicoverpa zea (Lepidoptera: Noctuidae) to Bt transgenic corn and cotton." Journal of Economic Entomology **96**(1): 173-187.

169. Storer, N. P., S. L. Peck, et al. (2003). "Spatial processes in the evolution of resistance in Helicoverpa zea (Lepidoptera: Noctuidae) to Bt transgenic corn and cotton in a mixed agroecosystem: a biology-rich stochastic simulation model." Journal of Economic Entomology **96**(1): 156-172.

170. Sutherst, R. W. and H. N. Comins (1979). "The management of acaricide resistance in the cattle tick, Boophilus microplus (Canestrini) (Acari: Ixodidae), in Australia." Bulletin of Entomological Research **69**(3): 519-540.

171. Tabashnik, B. E. (1986). "Evolution of pesticide resistance in predator/prey systems." Bulletin of the Entomological Society of America **32**(3): 156-161.

172. Tabashnik, B. E. (1986). "Model for managing resistance to fenvalerate in the diamondback moth (Lepidoptera: Plutellidae)." Journal of Economic Entomology **79**(6): 1447-1451.

173. Tabashnik, B. E. (1990). "Implications of gene amplification for evolution and management of insecticide resistance." Journal of Economic Entomology **83**(4): 1170-1176.

174. Tabashnik, B. E. (1994). "Delaying insect adaptation to transgenic plants - seed mixtures and refugia reconsidered." Proceedings of the Royal Society of London Series B-Biological Sciences **255**(1342): 7-12.

175. Tabashnik, B. E., T. J. Dennehy, et al. (2005). "Delayed resistance to transgenic cotton in pink bollworm." Proc Natl Acad Sci U S A **102**(43): 15389-93.

176. Tabashnik, B. E., F. Gould, et al. (2004). "Delaying evolution of insect resistance to transgenic crops by decreasing dominance and heritability." J Evol Biol **17**(4): 904-12; discussion 913-8.

177. Tang, S., Y. Xiao, et al. (2005). "Integrated pest management models and their dynamical behaviour." Bull Math Biol **67**(1): 115-35.

178. Tchetgen, E., E. H. Kaplan, et al. (2001). "Public health consequences of screening patients for adherence to highly active antiretroviral therapy." JAIDS, Journal of Acquired Immune Deficiency Syndromes **26**(2): 118-129.

179. Temime, L., P. Y. Boelle, et al. (2005). "Penicillin-resistant pneumococcal meningitis: high antibiotic exposure impedes new vaccine protection." Epidemiology and Infection **133**(3): 493-501.

180. Vacher, C., D. Bourguet, et al. (2003). "Modelling the spatial configuration of refuges for a

sustainable control of pests: a case study of Bt cotton." J Evol Biol **16**(3): 378-87.

181. Wahl, L. M. and M. A. Nowak (2000). "Adherence and drug resistance: predictions for therapy outcome." Proceedings of the Royal Society of London Series B-Biological Sciences **267**(1445): 835-843.

182. Webb, G. F., E. M. C. D'Agata, et al. (2005). "A model of antibiotic-resistant bacterial epidemics in hospitals." Proc Natl Acad Sci U S A **102**(37): 13343-8.

183. Weersink, A., R. S. Llewellyn, et al. (2005). "Economics of pre-emptive management to avoid weed resistance to glyphosate in Australia." Crop Protection **24**(7): 659-665.

184. Wijngaarden, P. J., F. van den Bosch, et al. (2005). "Adaptation to the cost of resistance: a model of compensation, recombination, and selection in a haploid organism." Proceedings of the Royal Society of London. Series B, Biological Sciences **272**(1558): 85-89.

185. Wodarz, D. and A. L. Lloyd (2004). "Immune responses and the emergence of drug-resistant virus strains in vivo." Proceedings of the Royal Society of London Series B-Biological Sciences **271**(1544): 1101-1109.

186. Xu, D. S., J. Curtis, et al. (2005). "On the role of schistosome mating structure in the maintenance of drug resistant strains." Bulletin of Mathematical Biology **67**(6): 1207-1226.

187. Yeung, S. M., W. Pongtavornpinyo, et al. (2004). "Antimalarial drug resistance, artemisinin-based combination therapy, and the contribution of modeling to elucidating policy choices." American Journal of Tropical Medicine and Hygiene **71**(2 supplement): 179-186.
