# Supplementary material for: Structure of the Scientific Community Modelling the Evolution of Resistance
Source: PLoS One. 2007 Dec 5;2(12):e1275. doi: 10.1371/journal.pone.0001275 (PMC2094735; doi:10.1371/journal.pone.0001275)
Supplement: Table S2 — Number of articles falling into the various descriptive categories for each group of the authorship network (0.03 MB PDF) [file pone.0001275.s002.pdf]

**Table S2**. Number of articles falling into the various descriptive categories for each group of the authorship network. For all categories, the distributions differ significantly between groups (Fisher exact test, $p < 10^{-5}$).

| Category | Descriptor | Number of Articles | | | |
|---|---|---|---|---|---|
| | | A1 Group | A2 Group | Small Groups | Isolated Articles |
| Type of Drug or Pesticide | Antibiotic Drug | 0 | 10 | 10 | 9 |
| | Antihelminthic Drug | 0 | 0 | 8 | 2 |
| | Antimalarial Drug | 0 | 1 | 6 | 5 |
| | Antiviral Drug | 0 | 3 | 6 | 5 |
| | Fungicide | 0 | 0 | 13 | 2 |
| | Herbicide | 0 | 0 | 15 | 3 |
| | Insecticidal Protein | 28 | 0 | 5 | 6 |
| | Insecticide | 12 | 0 | 4 | 14 |
| | Miticide | 1 | 0 | 2 | 0 |
| | Unspecific | 4 | 1 | 9 | 3 |
| Type of Target Organism | Farm Pest or Disease | 45 | 1 | 48 | 25 |
| | Human Parasite | 0 | 14 | 24 | 20 |
| | Unspecific | 0 | 0 | 6 | 4 |
| Modelling Approach | Epidemiology | 0 | 10 | 19 | 12 |
| | Population Genetics | 45 | 2 | 38 | 25 |
| | Other | 0 | 3 | 21 | 12 |
| First Author's Location | Africa | 0 | 0 | 1 | 0 |
| | Asia | 0 | 0 | 8 | 5 |
| | Europe | 1 | 5 | 35 | 17 |
| | North America | 43 | 10 | 25 | 21 |
| | Oceania | 1 | 0 | 9 | 3 |
| | South America | 0 | 0 | 0 | 3 |
| First Author's Discipline | Biology | 43 | 11 | 53 | 32 |
| | Economy | 0 | 0 | 0 | 1 |
| | Mathematics | 2 | 1 | 9 | 4 |
| | Medicine | 0 | 3 | 16 | 12 |
