# Supplementary material for: Structure of the Scientific Community Modelling the Evolution of Resistance
Source: PLoS One. 2007 Dec 5;2(12):e1275. doi: 10.1371/journal.pone.0001275 (PMC2094735; doi:10.1371/journal.pone.0001275)
Supplement: Table S3 — Multiresponse permutation procedure (MRPP) analysis of group dissimilarities showing mean citation distance between articles and mean source articles distance between citations in each citation group (0.02 MB PDF) [file pone.0001275.s003.pdf]

Table S3. Multiresponse permutation procedure (MRPP) analysis of group dissimilarities showing mean citation distance between articles and mean source articles distance between citations in each citation group. The chance-corrected agreement index (A) expresses the within-group homogeneity and has a maximum value of 1 when there is no dissimilarity among elements of any group. The *p*-value is the probability of obtaining by chance a value of A equal or larger than the observed value.

	Among Articles According to Their Citations					Among Citations According to Their Source Articles			
Citation Group	Group Size	Distance	A	*p*-value		Group Size	Distance	A	*p*-value
C1	138	0.98	0.010	$<10^{-3}$		631	0.98	0.013	$<10^{-3}$
C2	44	0.96				226	0.95		
