# Supplementary material for: Structure of the Scientific Community Modelling the Evolution of Resistance
Source: PLoS One. 2007 Dec 5;2(12):e1275. doi: 10.1371/journal.pone.0001275 (PMC2094735; doi:10.1371/journal.pone.0001275)
Supplement: Table S4 — Contingency table crossing for citation groups obtained by applying the clustering algorithm to the bipartite citation and to the unipartite article networks (0.01 MB PDF) [file pone.0001275.s004.pdf]

Table S4. Contingency table crossing for citation groups obtained by applying the clustering algorithm to the bipartite citation and to the unipartite article networks. The six first splits of this unipartite network separated small groups of articles (composed of one or two articles) from a larger network. The seventh split highlighted two clusters of almost equal size called U1 and U2.

Number of Articles		Bipartite Citation Network	
		C1	C2
Unipartite Articles Network	U1	128	7
	U2	5	41
	Small Groups	5	3
