# Supplementary material for: Structure of the Scientific Community Modelling the Evolution of Resistance
Source: PLoS One. 2007 Dec 5;2(12):e1275. doi: 10.1371/journal.pone.0001275 (PMC2094735; doi:10.1371/journal.pone.0001275)
Supplement: Table S5 — Contingency table crossing for citation groups obtained by applying the clustering algorithm to the bipartite citation and to the unipartite co-citation networks (0.01 MB PDF) [file pone.0001275.s005.pdf]

Table S5. Contingency table crossing for citation groups obtained by applying the clustering algorithm to the bipartite citation and to the unipartite co-citation networks. The unipartite co-citation network was formed of 16 isolated articles and a large connected component containing 171 papers. The first split of this large component yielded two large groups of papers called M1 and M2.

Number of Articles		Bipartite Citation Network		
		C1	C2	Isolated
Unipartite Co-citation Network	M1	122	0	0
	M2	11	38	0
	Isolated Articles	5	6	5
