# Supplementary material for: Structure of the Scientific Community Modelling the Evolution of Resistance
Source: PLoS One. 2007 Dec 5;2(12):e1275. doi: 10.1371/journal.pone.0001275 (PMC2094735; doi:10.1371/journal.pone.0001275)
Supplement: Table S6 — Number of articles falling into the various descriptive categories for each group of the citation network (0.03 MB PDF) [file pone.0001275.s006.pdf]

Table S6. Number of articles falling into the various descriptive categories for each group of the citation network. For all categories, the distributions differ significantly between groups (Fisher exact test, $p < 10^{-5}$).

Category	Descriptor	Number of Articles		
		C1 Group	C2 Group	Isolated Articles
Type of Drug or Pesticide	Antibiotic Drug	0	27	2
	Antihelminthic Drug	10	0	0
	Antimalarial Drug	12	0	0
	Antiviral Drug	0	14	0
	Fungicide	14	1	0
	Herbicide	18	0	0
	Insecticidal Protein	38	0	1
	Insecticide	29	0	1
	Miticide	2	0	1
	Unspecific	15	2	0
Type of Target Organism	Farm Pest or Disease	115	1	3
	Human Parasite	16	41	1
	Unspecific	7	2	1
Modelling Approach	Epidemiology	9	32	0
	Population Genetics	105	2	3
	Other	24	10	2
First Author's Location	Africa	0	0	1
	Asia	12	1	0
	Europe	39	17	2
	North America	72	25	2
	Oceania	13	0	0
	South America	2	1	0
First Author's Discipline	Biology	119	16	4
	Economy	0	1	0
	Mathematics	10	6	0
	Medicine	9	21	1
