# Supplementary material for: Structure of the Scientific Community Modelling the Evolution of Resistance
Source: PLoS One. 2007 Dec 5;2(12):e1275. doi: 10.1371/journal.pone.0001275 (PMC2094735; doi:10.1371/journal.pone.0001275)
Supplement: Table S7 — Multiresponse permutation procedure (MRPP) analysis of group dissimilarities showing mean citation distance between articles and mean source article distance between citations in each citation group (0.02 MB PDF) [file pone.0001275.s007.pdf]

Table S7. Multiresponse permutation procedure (MRPP) analysis of group dissimilarities showing mean citation distance between articles and mean source article distance between citations in each citation group. The chance-corrected agreement index (A) expresses the within-group homogeneity and has a maximum value of 1 when there is no dissimilarity between elements of a group. The *p*-value is the probability of obtaining by chance a value of A equal or larger than the observed value. Here the C1 cluster was split into 7 subgroups and the whole network therefore had 8 groups.

	Among Articles According to Their Citations					Among Citations According to Their Source Articles			
Citation group	Group Size	Distance	A	*p*-value		Group Size	Distance	A	*p*-value
C1, subgroup 1	13	0.92	0.058	$<10^{-3}$		53	0.85	0.081	$<10^{-3}$
C1, subgroup 2	18	0.94				88	0.89		
C1, subgroup 3	16	0.82				51	0.89		
C1, subgroup 4	10	0.89				28	0.82		
C1, subgroup 5	5	0.77				46	0.64		
C1, subgroup 6	11	0.89				104	0.84		
C1, subgroup 7	65	0.95				261	0.96		
C2	44	0.96				226	0.95		
