# Supplementary material for: Structure of the Scientific Community Modelling the Evolution of Resistance
Source: PLoS One. 2007 Dec 5;2(12):e1275. doi: 10.1371/journal.pone.0001275 (PMC2094735; doi:10.1371/journal.pone.0001275)
Supplement: Table S8 — Number of articles focusing on the different types of drug or pesticide for each subgroup of the C1 cluster (0.02 MB PDF) [file pone.0001275.s008.pdf]

Table S8. Number of articles focusing on the different types of drug or pesticide for each subgroup of the C1 cluster. The distribution differs significantly between groups (Fisher exact test, $p < 10^{-5}$).

Descriptor	Number of Articles						
	C1 sub1	C1 sub2	C1 sub3	C1 sub4	C1 sub5	C1 sub6	C1 sub7
Antibiotic Drug	0	0	0	0	0	0	0
Antihelminthic Drug	0	0	0	9	0	0	1
Antimalarial Drug	12	0	0	0	0	0	0
Antiviral Drug	0	0	0	0	0	0	0
Fungicide	0	0	14	0	0	0	0
Herbicide	0	18	0	0	0	0	0
Insecticidal Protein	0	0	0	0	5	8	25
Insecticide	1	0	0	0	0	2	26
Miticide	0	0	0	0	0	0	2
Unspecific	0	0	2	1	0	1	11
