# Supplementary material for: Structure of the Scientific Community Modelling the Evolution of Resistance
Source: PLoS One. 2007 Dec 5;2(12):e1275. doi: 10.1371/journal.pone.0001275 (PMC2094735; doi:10.1371/journal.pone.0001275)
Supplement: Table S9 — List of the 48 references cited by articles belonging to the C1 group and articles belonging to the C2 group (0.02 MB PDF) [file pone.0001275.s009.pdf]

Table S9. List of the 48 references cited by articles belonging to the C1 group and articles belonging to the C2 group.

1. Anderson, R. M., and R. M. May. 1991. Infectious Diseases of Humans: Transmission and Control, 1 ed. Oxford University Press, Oxford, UK.
2. Anderson, R. M., and R. M. May. 1992. Infectious Diseases of Humans: Transmission and Control, 2 ed. Oxford University Press, Oxford, UK.
3. Austin, D. J., K. G. Kristinsson, and R. M. Anderson. 1999. The relationship between the volume of antimicrobial consumption in human communities and the frequency of resistance. Proceedings of the National Academy of Science USA 96:1152-6.
4. Austin, D. J., N. J. White, and R. M. Anderson. 1998. The dynamics of drug action on the within-host population growth of infectious agents: Melding pharmacokinetics with pathogen population dynamics. Journal of Theoretical Biology 194:313-339.
5. Baquero, F., and J. Blazquez. 1997. Evolution of antibiotic resistance. Trends in Ecology & Evolution 12:482-487.
6. Barrett, J. A. 1983. Estimating relative fitness in plant parasites: some general problems. Phytopathology 73:510-512.
7. Birch, C. P. D., and M. W. Shaw. 1997. When can reduced doses and pesticide mixtures delay the build-up of pesticide resistance? A mathematical model. Journal of Applied Ecology 34:1032-1042.
8. Blower, S. M., P. M. Small, and P. C. Hopewell. 1996. Control strategies for tuberculosis epidemics: New models for old problems. Science 273:497-500.
9. Bonhoeffer, S., M. Lipsitch, and B. R. Levin. 1997. Evaluating treatment protocols to prevent antibiotic resistance. Proceedings of the National Academy of Science USA 94:12106-11.
10. Bonhoeffer, S., and M. A. Nowak. 1997. Pre-existence and emergence of drug resistance in HIV-1 infection. Proceedings of the Royal Society of London B 264:631-7.
11. Bremermann, H. J., and H. M. Thieme. 1989. A competitive-exclusion principle for pathogen virulence. Journal of Mathematical Biology 27:179-190.
12. Chin, K. M. 1987. A simple method of selection for fungicide resistance in plant pathogen populations. Phytopathology 77:666-669.
13. Crow, J. F., and M. Kimura. 1970. An introduction to population genetics theory. Harper & Row, New York, USA.
14. Dekker, J. 1976. Acquired resistance to fungicides. Annual Review of Phytopathology 14:511 pp.
15. Durrett, R., and S. Levin. 1993. The importance of being discrete (and spatial). Theoretical Population Biology 46:363-394.
16. Georghiou, G. P. 1986. Pesticide resistance: strategies and tactics for management. National Academy Press, Washington, DC USA.
17. Gubbins, S., and C. A. Gilligan. 1999. Invasion thresholds for fungicide resistance: deterministic and stochastic analyses. Proceedings of the Royal Society of London B 266:2539-2549.
18. Holt, R. D. 1977. Predation, apparent competition and the structure of the prey community. Theoretical Population Biology 12:197-229.
19. Kable, P. F., and H. Jeffery. 1980. Selection for tolerance in organisms exposed to sprays of biocide mixtures: a theoretical model. Phytopathology 70:8-12.
20. Karaoglanidis, G. S., C. C. Thanassoulopoulos, and P. M. Ioannidis. 2001. Fitness of Cercospora beticola field isolates - resistant and - sensitive to demethylation inhibitor fungicides. European Journal of Plant Pathology 107:337-347.

21. Kermack, W. O., and A. G. McKendrick. 1927. A contribution to the mathematical theory of epidemics. Proceedings of the Royal Society of London A 115:700-721.
22. Leonard, K. J., and R. J. Czochor. 1980. Theory of genetic interactions among populations of plants and their pathogens. Annual Review of Phytopathology 18:237-258.
23. Levin, B. R., and R. M. Anderson. 1999. The population biology of anti-infective chemotherapy and the evolution of drug resistance: more questions than answers, p. 125-137, Evolution in Health and Disease. Oxford University Press.
24. Levy, Y., R. Levi, and Y. Cohen. 1983. Build-up of a pathogen subpopulation resistant to a systemic fungicide under various control strategies: a flexible simulation model. Phytopathology 73:1475-1480.
25. Lipsitch, M., C. T. Bergstrom, and B. R. Levin. 2000. The epidemiology of antibiotic resistance in hospitals: paradoxes and prescriptions. Proceedings of the National Academy of Science USA 97:1938-43.
26. Lipsitch, M., and B. R. Levin. 1997. The population dynamics of antimicrobial chemotherapy. Antimicrob Agents Chemother 41:363-73.
27. May, R. M., and R. M. Anderson. 1979. Population biology of infectious diseases: Part II. Nature 280:455-461.
28. May, R. M., and A. P. Dobson. 1986. Population dynamics and the rate of pesticide evolution, National Research Council, Pesticide resistance. National Academy of Science, Washington.
29. McPeek, M. A., and R. D. Holt. 1992. The evolution of dispersal in spatially and temporally varying environments. American Naturalist 140:1010-1027.
30. Milgroom, M. G. 1990. A stochastic model for the initial occurrence and development of fungicide resistance in plant pathogen populations. Phytopathology 80:410-416.
31. Milgroom, M. G., S. A. Levin, and W. E. Fry. 1989. Population genetics theory and fungicide resistance, p. xii + 377 pp. In K. J. Leaonard and G. E. Fry (ed.), Plant disease epidemiology Vol. 2: genetics, resistance, and management. MacGraw-Hill Publishing Co., New York.
32. Nowak, M. A., and R. M. May. 1994. Superinfiection and the evolution of parasite virulence. Proceedings of the Royal Society of London B 255:81-89.
33. Peck, S. L., and S. P. Ellner. 1997. The effect of economic thresholds and life-history parameters on the evolution of pesticide resistance in a regional setting. American Naturalist 149:43-63.
34. Phillips, M., and P. A. Phillips-Howard. 1996. Economic implication of resistance to antimalarial drugs. Pharmacoeconomics 10:225-238.
35. Press, W. H., B. P. Flannery, S. A. Teukolsky, and W. T. Vetterling. 1992. Numerical Recipes in C, 2 ed. Cambridge University Press, Cambridge, UK.
36. Rausher, M. D. 2001. Co-evolution and plant resistance to natural enemies. Nature 411:857-864.
37. Renshaw, E. 1991. Modelling biological poulations in space and time. Cambridge University Press, Cambridge, UK.
38. Ribeiro, R. M., S. Bonhoeffer, and M. A. Nowak. 1998. The frequency of resistant mutant virus before antiviral therapy. Aids 12:461-465.
39. Rosenheim, J. A., and B. E. Tabashnik. 1991. Influence of generation time on the rate of response to selection. American Naturalist 137:527-541.
40. Shaw, M. W. 1989. Independent action of fungicides and its consequences for strategies to retard the evolution of fungicide resistance. Crop Protection 8:405-411.
41. Shaw, M. W. 1989. A model of the evolution of polygenically controlled fungicide resistance. Plant Pathology 38:44-55.

42. Shaw, M. W. 1993. Theoretical analysis of the effect of interacting activities on the rate of selection for combined resistance to fungicide mixtures. Crop Protection 12:120-126.
43. Shigesada, N., and K. Kawasaki. 1997. Biological invasions: theory and practice. Oxford University Press, Oxford UK.
44. Skylakakis, G. 1982. Epidemiological factors affecting the rate of selection of biocide-resistant genotypes of plant pathogenic fungi. Phytopathology 72:271-273.
45. Skylakakis, G. 1980. Estimating parasitic fitness of plant pathogenic fungi: a theoretical contribution. Phytopathology 70:696-698.
46. Warwick, S. I. 1991. Herbicide resistance in weedy plants: physiology and population biology. Annual Review of Ecology and Systematics 22:95-114.
47. Williamson, M. 1972. The analysis of biological populations. Edward Arnold, London, UK.
48. Wilson, E. O. 1971. The insect societies. Harvard University Press, Cambridge, Mass., USA.
