# Supplementary material for: Structure of the Scientific Community Modelling the Evolution of Resistance
Source: PLoS One. 2007 Dec 5;2(12):e1275. doi: 10.1371/journal.pone.0001275 (PMC2094735; doi:10.1371/journal.pone.0001275)
Supplement: Table S10 — Characteristics of the 28 articles of one group that cited references of the other group (0.02 MB PDF) [file pone.0001275.s010.pdf]

Table S10. Characteristics of the 28 articles of one group that cited references of the other group.

Descriptor	Number of Articles		Percentage of Papers for Each Class in the Database	
	C1 Group	C2 Group	C1 Group	C2 Group
Antibiotic Drug	0	4	0	13.8
Antihelminthic Drug	0	0	0	0
Antimalarial Drug	6	0	50	0
Antiviral Drug	0	0	0	0
Fungicide	4	1	26.7	6.7
Herbicide	3	0	16.7	0
Insecticidal Protein	3	0	7.7	0
Insecticide	2	0	6.7	0
Miticide	0	0	0	0
Unspecific	3	2	17.6	11.8
