# Supplementary material for: Structure of the Scientific Community Modelling the Evolution of Resistance
Source: PLoS One. 2007 Dec 5;2(12):e1275. doi: 10.1371/journal.pone.0001275 (PMC2094735; doi:10.1371/journal.pone.0001275)
Supplement: Table S11 — Formulae used to search for relevant articles describing models of the evolution of resistance to pesticides and drugs within the CABs (1973–2006), Current Contents (1998–2006), and Medline (1950–2006) databases (0.02 MB PDF) [file pone.0001275.s011.pdf]

Table S11. Formulae used to search for relevant articles describing models of the evolution of resistance to pesticides and drugs within the CABs (1973-2006), Current Contents (1998-2006), and Medline (1950-2006) databases. The 'final formula' for each database search consisted in the combination of the common and the database-specific formulae. Thus, the 'Number of hits' column presents the results of these combinations. This search provided a total dataset of 1,894 non redundant references (see Table S1).

Type of Formula	Database	Timespan	Type of Descriptors	Formula of Descriptors	Number of hits
Common	All	1973-2006	Topics	(insecticid* OR fungicid* OR herbicid* OR nematicid* OR acaricid* OR rodenticid* OR miticid* OR pesticid* OR antibio* OR antivir* OR bacillus) AND (resistan*) AND (model*)	-
Specific	CABs	1973-2006	Cabicodes	((HH000 or HH400 or HH405 or HH410 or FF500 or FF600) and DE=models)*	817
	Current Contents	1998-2006	Scientific area codes restriction	((Plant Sciences) OR (Multidisciplinary) OR (Experimental Biology) OR (Biology) OR (Molecular Biology Genetics) OR (Entomology Pest Control) OR (Research Laboratory Medicine Medical Technology) OR (Medical Research General Topics) OR (Immunology))	808
	Medline	1950-2006	MeSH	"Drug Resistance"[MAJR] OR "Drug Tolerance"[MAJR] OR "Models, Theoretical"[MAJR] OR "Computer Simulation"[MAJR] OR "Mathematical Computing"[MAJR] OR "Evolution"[MAJR] OR "Adaptation, Biological"[MAJR]	360

* Codes used for the Cabicodes:
H000: Pathogen, Pest and Parasite and Weed Management (General)
HH400: Pesticides and Drugs (General)
HH405: Pesticides and Drugs: Control
HH410: Pesticide and Drug Resistance
FF500: Weeds and Noxious Plants
FF600: Pests, Pathogens and Biogenic Diseases of Plants.
